# Feasibility of Short-Term Aggressive Lipid-Lowering Therapy with the PCSK9 Antibody in Acute Coronary Syndrome

**DOI:** 10.3390/jcdd10050204

**Published:** 2023-05-09

**Authors:** Satoshi Yamashita, Atsushi Sakamoto, Satoshi Shoji, Yoshitaka Kawaguchi, Yasushi Wakabayashi, Masaki Matsunaga, Kiyohisa Suguro, Yuji Matsumoto, Hiroyuki Takase, Tomoya Onodera, Kei Tawarahara, Masahiro Muto, Yasutaka Shirasaki, Hideki Katoh, Makoto Sano, Kenichiro Suwa, Yoshihisa Naruse, Hayato Ohtani, Masao Saotome, Tsuyoshi Urushida, Shun Kohsaka, Eisaku Okada, Yuichiro Maekawa

**Affiliations:** 1 Division of Cardiology, Internal Medicine III, Hamamatsu University School of Medicine, Hamamatsu 4313192, Japan; sassy.y.0209.1031.1216@gmail.com (S.Y.); asakamo@hama-med.ac.jp (A.S.); makosano@hama-med.ac.jp (M.S.); k-suwa@hama-med.ac.jp (K.S.); ynaruse@hama-med.ac.jp (Y.N.); ohtani@hama-med.ac.jp (H.O.); msaotome@hama-med.ac.jp (M.S.); uru173jrc@gmail.com (T.U.); 2 Department of Cardiology, Hino Municipal Hospital, Hino 1910062, Japan; sshoji0116@gmail.com; 3 Department of Cardiology, Seirei Mikatahara Hospital, Hamamatsu 4338558, Japan; y.kawaguchi@sis.seirei.or.jp (Y.K.); ywakabayashi@sis.seirei.or.jp (Y.W.); 4 Department of Cardiology, Iwata City Hospital, Iwata 4388550, Japan; masaki@hospital.iwata.shizuoka.jp; 5 Department of Cardiology, Fujinomiya City Hospital, Fujinomiya 4180076, Japan; fujinomiya_suguro@hospital.fujinomiya.shizuoka.jp; 6 Department of Cardiology, Kikugawa City Hospital, Kikugawa 4390022, Japan; yumat3@kikugawa-hosp.jp; 7 Department of Internal Medicine, Enshu Hospital, Hamamatsu 4300929, Japan; h-takase.ken@shizuokakouseiren.jp; 8 Department of Cardiology, Shizuoka City Shizuoka Hospital, Shizuoka 4208630, Japan; bravetomtom@yahoo.co.jp; 9 Department of Cardiology, Hamamatsu Red Cross Hospital, Hamamatsu 4348533, Japan; tkei1220@gmail.com; 10 Department of Cardiology, Hamamatsu Medical Center, Hamamatsu 4328580, Japan; m-mutou@hmedc.or.jp; 11 Shirasaki Clinic, Kuki 3460031, Japan; yasutaka.shirasaki@shirasaki-heart.jp; 12 Department of Cardiology, Kosai General Hospital, Kosai 4310431, Japan; hkatoh56@gmail.com; 13 Department of Cardiology, Keio University School of Medicine, Tokyo 1608582, Japan; sk@keio.jp; 14 Department of Faculty of Social Policy and Administration, Hosei University, Tokyo 1028160, Japan; eisaku.okada0129@gmail.com

**Keywords:** PCSK9 antibody, acute coronary syndrome, lipid-lowering therapy, low-density lipoprotein cholesterol

## Abstract

Background: The guideline-recommended low-density lipoprotein cholesterol target level of <70 mg/dL may not be achieved with statin administration in some patients with acute coronary syndrome (ACS). Therefore, the proprotein convertase subtilisin-kexin type 9 (PCSK9) antibody can be added to high-risk patients with ACS. Nevertheless, the optimal duration of PCSK9 antibody administration remains unclear. Methods and Results: Patients were randomized to receive either 3 months of lipid lowering therapy (LLT) with the PCSK9 antibody followed by conventional LLT (with-PCSK9-antibody group) or 12 months of conventional LLT alone (without-PCSK9-antibody group). The primary endpoint was the composite of all-cause death, myocardial infarction, stroke, unstable angina, and ischemia-driven revascularization. A total of 124 patients treated with percutaneous coronary intervention (PCI) were randomly assigned to the two groups (n = 62 in each). The primary composite outcome occurred in 9.7% and 14.5% of the patients in the with- and without-PCSK9-antibody groups, respectively (hazard ratio: 0.70; 95% confidence interval: 0.25 to 1.97; *p* = 0.498). The two groups showed no significant differences in hospitalization for worsening heart failure and adverse events. Conclusions: In ACS patients who underwent PCI, short-term PCSK9 antibody therapy with conventional LLT was feasible in this pilot clinical trial. Long-term follow-up in a larger scale clinical trial is warranted.

## 1. Introduction

Low density lipoprotein cholesterol (LDL-C) levels are important for secondary prevention of acute coronary syndrome (ACS) [1,2]. Lipid-lowering therapy (LLT) with high-dose statins is effective [3] and is the first line of treatment. However, in actual clinical practice, LDL-C levels are often not lowered to the target level of <70 mg/dL [2,4,5], necessitating the addition of ezetimibe and a human monoclonal antibody to proprotein convertase subtilisin-kexin type 9 (PCSK9) and other agents to reduce the risk of cardiovascular (CV) events [6,7,8]. The PCSK9 antibody activates low-density lipoprotein receptor recycling by inhibiting PCSK9 binding to low-density lipoprotein receptors. It has been shown to have potent effects in reducing LDL-C levels, decreasing CV events [8,9], and inducing plaque regression [10,11]. The concept of metabolic memory or legacy effect in the field of diabetes mellitus posits that the effect of controlling blood glucose cooperatively for a certain period of time will have a long-lasting effect and eventually have a positive impact on long-term prognosis [12,13]. The same concept has been validated for LLTs in the past. The 20-Year follow-up of the West of Scotland Coronary Prevention Study demonstrated that statin treatment for 5 years was associated with a legacy benefit, along with improved survival and a substantial reduction in CV disease outcomes over a 20-year period, supporting the wider adoption of primary prevention strategies [14]. A similar study reported that good initial lipid control with statin therapy can have a legacy effect [15]. However, despite the strong need for aggressive LLT, long-term adherence to PCSK9 antibody administration may be compromised, as it is a high-cost drug that requires administration by injection every 2–4 weeks [16]. Although the development of a human monoclonal antibody to PCSK9 has made it easier to achieve good lipid control, its effect on long-term prognosis remains unclear [17]. Thus, in this study, we investigated the feasibility of introducing PCSK9 antibodies administered as early as possible and for a limited period of time (3 months) after percutaneous coronary intervention (PCI) in patients with ACS in comparison with conventional LLT.

## 2. Materials and Methods

### 2.1. Study Design

The Short-Term Aggressive Lipid-loweRing Therapy with the PCSK9 (START-PCSK9) (UMIN000030860) (date of first trial registration: 1 March 2018) was a multicenter, prospective, randomized, and controlled study conducted in 9 hospitals and 1 clinic.

### 2.2. Participants

Participants were enrolled between March 2018 and May 2020. START-PCSK9 enrolled patients with ACS who underwent PCI and whose LDL-C levels were 70 mg/dL or higher. Participants were eligible if they were aged 20 years or older and diagnosed with ACS, which included ST elevation myocardial infarction (MI), non-ST elevation MI, and unstable angina. The main exclusion criteria were age <20 years, PCSK9 antibody treatment prior to hospitalization, renal insufficiency with a serum creatinine level >3.0 mg/dL, or acute or chronic liver disease defined by serum levels of transaminases >3 times the upper limit of normal at screening.

### 2.3. Randomization and Masking

Participants were randomly assigned in a 1:1 ratio to receive treatment with conventional LLT and PCSK9 antibody administration (with-PCSK9-antibody group) or conventional LLT alone (without-PCSK9-antibody group). The patients in the with-PCSK9-antibody group received evolocumab at a dose of 140 mg every 2 weeks or 420 mg every 4 weeks, alirocumab at a dose of 75 mg every 2 weeks, or 150 mg every 4 weeks. Consecutive allocation of randomization codes to individual participants was concealed using a web-based (interactive web response system) randomization system. Although the study was conducted using an open-label system, data were masked from the statistician until the database was released. Independent data reviewers monitored aggregate safety data. The primary and second endpoint analyses were conducted in the intention-to-treat population, which included all patients who underwent randomization. None of the patients discontinued the treatment in either of the 2 groups during the study period. Conventional LLT was defined as follows. First, use of high-intensity statins to the extent possible was considered but if not possible, moderate-intensity statin therapy could be initiated. The first goal was to achieve LDL-C levels of 70 mg/dL, but if the LDL-C levels remained >70 mg/dL on maximally tolerated statin therapy, adding ezetimibe was possible. The choice of which statin to use in this study was at the discretion of the investigators. After 3 months of PCSK9 antibody administration in the with-PCSK9-antibody group, no PCSK9 antibody was to be used in either group. Follow-up evaluations were conducted at 30 days (within a window of ±7 days) and 90 days (±7-day window) after randomization exclusively in on-site visits and at 360 days (±60-day window) after randomization exclusively in an on-site visit. Follow-up data were obtained from outpatient consultations, blood tests, electrocardiography, and standardized questionnaires.

### 2.4. Outcomes

The primary endpoint was defined as the composite of all-cause death, MI, stroke, unstable angina, and ischemia-driven revascularization. Ischemia-driven revascularization was defined as ischemic symptoms consistent with Canadian cardiovascular society class ≥2 angina despite guideline-based medical therapy and at least 1 of the following [18]:

(1) A positive functional study such as an exercise or Persantine myocardial perfusion imaging, a stress or dobutamine echo, or other imaging demonstrating clear evidence of reversible ischemia corresponding to stenosis.
(2) New ischemic electrocardiographic changes consistent with stenosis.
(3) A fractional flow reserve ≤ 0.80.

Secondary endpoints included (1) hospitalization for worsening heart failure and time to occurrence and (2) adverse events, including myalgia and laboratory test abnormalities, defined based on elevation of the levels of alanine aminotransferase, aspartate aminotransferase, or both to ≥3 times the upper limit of normal and elevation of the levels of creatine kinase (CK) to ≥5 times the upper limit of normal. Adverse events referred to events that developed after the start of the assigned treatment and for which a causal relationship to the study drug administration could not be ruled out. Adverse events were assessed and reported by the site investigators. Exploratory endpoints included changes in serum LDL-C, high-density lipoprotein cholesterol (HDL-C), and triglyceride levels. We expected patient enrollment to last 1 year. In the previous study that enrolled Japanese patients with ACS, the cumulative incidence of all-cause death, non-fatal MI, non-fatal stroke, unstable angina, or revascularization with either PCI or coronary artery bypass graft, was approximately 25.0% for intensive treatment at 1 year [3]. Considering the possibility of similar-risk patients being enrolled in our study, we assumed an event rate of 25.0% at 1 year. An initial sample size was selected to afford 80% power to detect relative risk reduction of 15% in the primary endpoint by a log-rank test at a significance level of 0.05. Given potential dropouts during follow up, 150 patients were planned to be enrolled. However, because of slow patient enrollment owing to the coronavirus disease pandemic, patient enrollment was prematurely terminated in May 2020, after enrolling 127 patients in 24 months.

### 2.5. Statistical Analysis

Continuous variables are expressed as mean ± standard deviation. Between-group comparisons were performed using an unpaired *t*-test or the Mann–Whitney U test. All categorical variables are presented as numbers and percentages for each group and were compared using the chi-square test or Fisher’s exact test. The primary and secondary endpoints were assessed by the time to the first occurrence of any event; the intention-to-treat principle was used, and event data for all patient data were included. The Kaplan–Meier method was used to evaluate time-to-event data for all-cause death, MI, stroke, unstable angina, ischemia-driven revascularization, and sustained clinical improvement over the 1-year follow-up period. Differences between groups were assessed using the log-rank test and hazard ratios (HRs) with 95% confidence intervals (CIs) in the Cox proportional-hazards model. To assess the effect of short-term PCSK9 antibody therapy across prespecified subgroups, subgroup-by-treatment interaction terms were added to the models. Statistical significance was set at *p* < 0.05. All statistical analyses were performed using IBM SPSS Statistics 25 (IBM, New York, NY, USA) and EZR (Saitama Medical Center, Jichi Medical University, Saitama, Japan), which is a graphical user interface for R (R Foundation for Statistical Computing, Vienna, Austria, version 3.3.2).

## 3. Results

### 3.1. Eligible Patients and Baseline Characteristics

**Figure 1:**
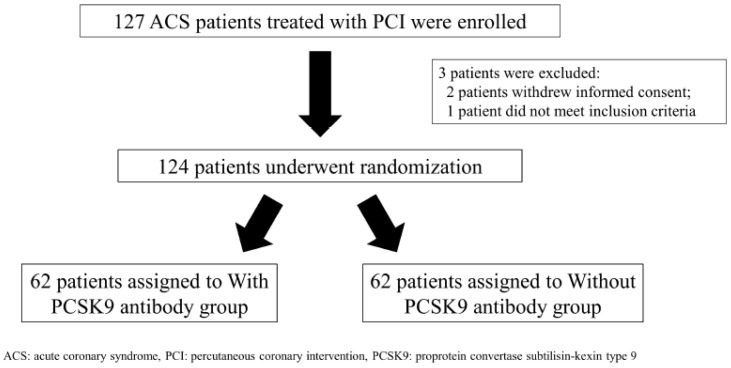
Scheme of patient randomization.

Between May 2018 and May 2020, 127 patients were enrolled in the study (Figure 1).

Of these, three patients were not randomized, and their data were excluded from the analysis. Two of these patients withdrew informed consent, and one patient was not eligible. All randomized patients underwent PCI. All patients in the with-PCSK9-antibody group were administered the PCSK9 antibody for a defined period of 3 months. The characteristics of the patients in the with- and without-PCSK9-antibody groups were well balanced (Table 1).

ST elevation MI was observed in 75% of the enrolled ACS patients. The time from PCI for ACS to randomization was 11.1 ± 16.6 days. In the with-PCSK9-antibody group, 64% and 36% of the patients received evolocumab and alirocumab, respectively. LDL-C levels at enrollment were 113.2 ± 32.8 mg/dL in the with-PCSK9 group and 111.3 ± 30.3 mg/dL in the without-PCSK9 group. Table 2 shows the procedural characteristics. The mean duration of follow up was 368 ± 38 days.

### 3.2. Primary and Secondary Endpoints

**Figure 2:**
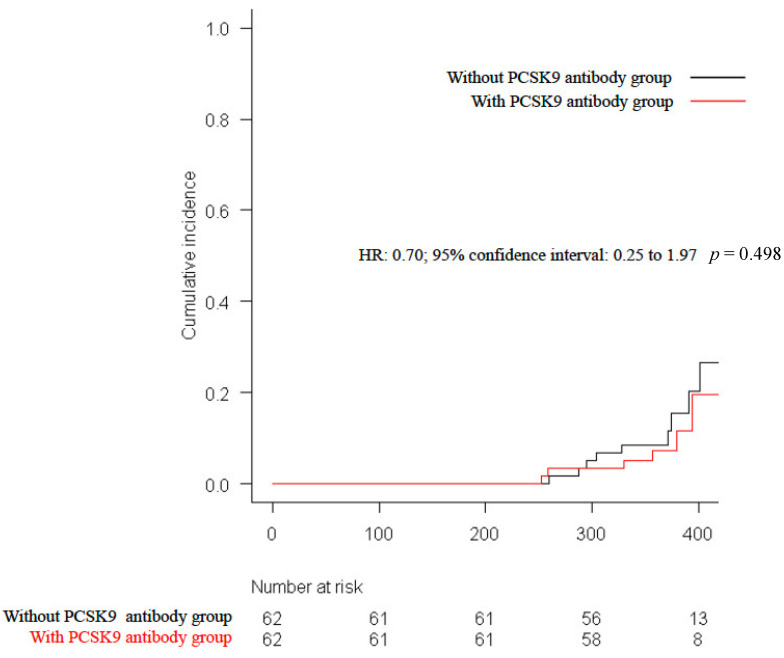
Kaplan–Meier rates of the primary endpoint at 1-year follow up.

**Figure 3:**
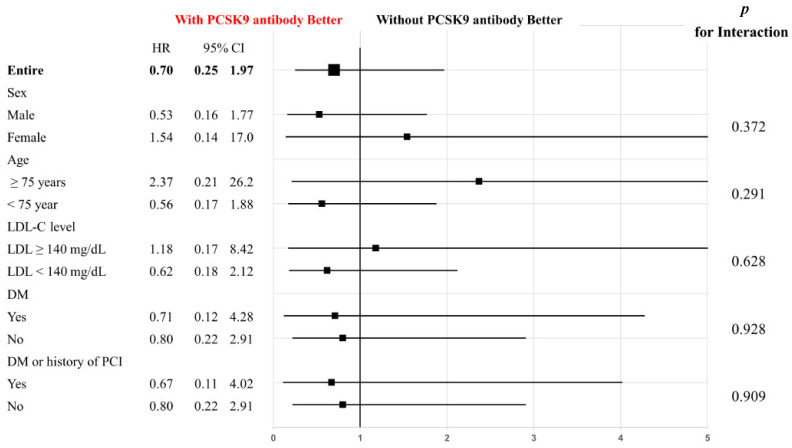
Subgroup analyses of the primary composite endpoint in pre-specified subgroups of the study cohort.

The primary endpoint of CV events was observed in six patients (9.7%) in the with-PCSK9-antibody group and in nine patients (14.5%) in the without-PCSK9-antibody group (HR: 0.70; 95% CI: 0.25 to 1.97; *p* = 0.498, Figure 2). Sex, age, LDL-C levels, presence of diabetes mellitus, and history of PCI were subjected to subgroup analyses for the primary endpoint, and there were no significant differences between the study groups (Figure 3).

Kaplan–Meier survival curves compared conventional lipid-lowering therapy with and without 3 months of PCSK9 antibody administration for the composite of all-cause death, myocardial infarction, stroke, unstable angina, and ischemia-driven revascularization. PCSK9, proprotein convertase subtilisin-kexin type 9.

Pre-specified subgroups included sex, age, low-density lipoprotein cholesterol levels, diabetes mellitus, and history of PCI. P interaction represents the likelihood of interaction between the variable and the treatment strategy (with PCSK9 antibody vs. without PCSK9 antibody). 

There were no differences in the secondary endpoint of hospitalization for worsening heart failure between the groups (HR: 0.98; 95% CI: 0.20 to 4.76; *p* = 0.980). There were no significant differences in liver dysfunction, serum CK value abnormalities, or the occurrence of myalgia (Table 3). Other potential adverse events which were not categorized in primary and secondary outcomes were listed in the Supplemental Table S1.

### 3.3. Time Course of Lipid Levels

**Figure 4:**
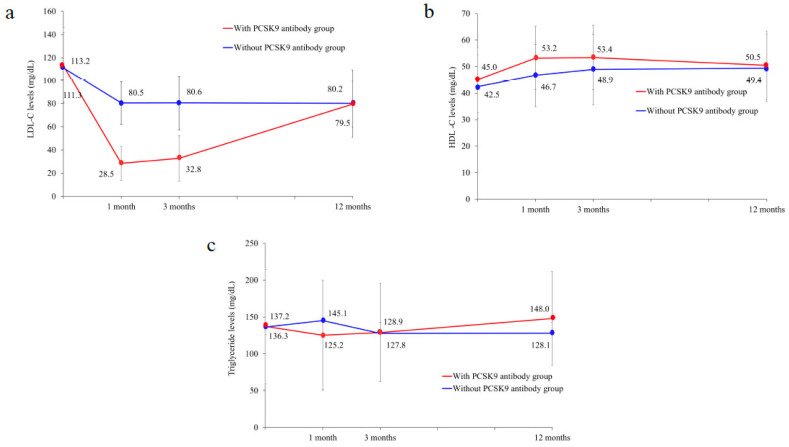
Changes in lipid parameters over time. (**a**–**c**): changes over time in low-density lipoprotein cholesterol, high-density lipoprotein cholesterol, and triglyceride levels.

LDL-C levels were significantly lower at 1 and 3 months in the with-PCSK9-antibody group than in the without-PCSK9-antibody group, but at 1 year they were similar and >70 mg/dL (Figure 4a, Table 4). HDL-C levels were significantly higher in the with-PCSK9-antibody group than in the without-PCSK9-antibody group at 1 month (Figure 4b, Table 4). There were no significant differences in triglyceride levels between the two groups during the observation period (Figure 4c, Table 4).

## 4. Discussion

In this study, we evaluated the feasibility of introducing PCSK9 antibody therapy for 3 months in patients treated with PCI for ACS. The two groups showed no significant difference in the primary endpoint of CV events (all-cause death, MI, stroke, unstable angina, and ischemia-driven revascularization). The secondary endpoints and safety outcomes were also similar between the two groups.

The European Society of Cardiology/European Atherosclerosis Society guidelines recommend considering the early addition of a PCSK9 inhibitor for the management of dyslipidemia in patients who present with ACS and have elevated LDL-C levels despite treatment with ezetimibe and a maximally tolerated dose of statins [17]. On the other hand, in terms of cost-effectiveness, lifelong administration of the PCSK9 antibody to all patients who present with ACS should be avoided, and we considered a study to limit the duration of PCSK9 antibody administration so that it could be administered to as many patients as possible. This study demonstrated that short-term administration of the PCSK9 antibody was associated with no safety concerns and that the LDL-C level remained consistently below 40 mg/dL for 3 months. Although the incidence of major adverse cardiac events was similar between the two groups, the short observation period and sample size may have influenced the results. As with previous studies regarding metabolic memory, long-term follow up is essential to evaluate the effect of 3-month administration of the PCSK9 antibody, despite the results of this interim analysis. In this study, patients in the with-PCSK9-antibody group were administered the PCSK9 antibody as early as possible after PCI. The time from PCI for ACS to randomization was 11.1 days. Thus, aggressive LLT was practiced earlier in this study. Moreover, all patients underwent PCI. These factors distinguished our study from the ODYSSEY outcome trial, in which the interval from the qualifying ACS to randomization was 2.6 months and approximately 70% of patients underwent PCI or coronary artery bypass grafting [9].

In the with-PCSK9 group of this study, the PCSK9 antibody was introduced in addition to statins early after the onset of ACS, and a significant decrease in the LDL-C level was observed at 1 and 3 months. ACS patients are known to show a high incidence of recurrent events in the early post-onset period. Therefore, reducing the LDL-C levels in this period inhibits early recurrent events [19,20,21]. Several clinical trials tested the effect of early PCSK9 inhibitor introduction by adding on statins during ACS hospitalization and proved the feasibility regarding early management of serum lipid profiles such as LDL-C and LP(a) levels [22,23]. Although the long-term solid clinical benefit of early PCSK9 inhibition in ACS patients need to be further proven by larger-scale clinical trials, recent studies using coronary imaging modality (i.e., optical coherence tomography) revealed that the addition of a PCSK9 inhibitor to statin therapy increased fibrous cap thickness and regressed lipid-rich plaques which are the surrogate of coronary plaque instability [24,25]. In the present study, administration of the PCSK9 antibody significantly reduced the LDL-C levels at an early stage and may indicate similar results.

Since the long-term effect of the PCSK9 antibody is unclear, we administered the PCSK9 antibody for a short period of time [8,9]. The American College of Cardiology/American Heart Association and European Society of Cardiology guidelines recommend prior administration of ezetimibe for cost-effectiveness [17,26]. This is also relevant because of the cost considerations that are associated with the introduction of the PCSK9 antibody [16,27,28]. Temporary administration of PCSK9 agents has been noted to cause regression of plaques in the coronary arteries [29] and, similar to statins, this may lead to legacy effects of the PCSK9 antibody.

## 5. Study Limitations

There are several limitations in our study which deserve attention. First, although the cost-effectiveness of treatment may differ between East Asia and Western countries, financial concerns regarding PCSK9 antibody treatment in ACS patients may be addressed given the benefits of short-term administration found in this study. Second, because of the slow inclusion of participants due to the current COVID-19 pandemic, the number of patients did not reach that derived from a pre-specified power calculation. Therefore, the result led in this study need to be interpreted with caution. Third, since this is a small-scale pilot study in line with real world clinical situation, due to the lack of enough number of participants subgroup analysis cannot be conducted in terms of three different protocols of PCSK9 antibody administration as well as different types of statins and other medications. Fourth, because our study had been designed as an open label, not a double-blind clinical trial, an unavoidable potential bias existed on both the sides of the study participants and physicians. This limitation needs to be overcome in future studies with a double-blind design using sham infusions in the control group. Finally, since the observation period in this study was short, further progress monitoring is necessary. Further large-scale, double blind, long-term prospective observational studies needed to be conducted for evaluating the potential effects on clinical outcomes.

## 6. Conclusions

In ACS patients who underwent PCI, there were no significant differences in CV events at 1 year between the groups treated with and without PCSK9 antibodies. There was a significant reduction in LDL-C during treatment in the with-PCSK9-antibody group. To our knowledge, this was the first study to examine the feasibility of introducing short-term limited administration of PCSK9 antibodies (i.e., three months of post-ACS period), and it adds to the debate on long-term adherence to PCSK9 antibody administration. No safety concerns were noted during the study; however, long-term follow-up in the context of a large-scale clinical trial is recommended.

## Figures and Tables

**Table 1:** Baseline patient characteristics.

	With PCSK9 Antibody Group (N = 62)	Without PCSK9 Antibody Group (N = 62)
Male	48 (77)	48 (77)
Age, years	66.9 ± 10.2	66.0 ± 11.6
BMI, kg/m^2^	24.2 ± 3.9	24.0 ± 3.6
Hypertension	43 (69)	39 (63)
Hyperlipidemia	49 (79)	47 (76)
Familial hyperlipidemia	2 (3)	1 (2)
Diabetes mellitus	20 (32)	21 (34)
Hyperuricemia	11 (18)	15 (24)
COPD	4 (6)	5 (8)
PAD	0 (0)	3 (5)
AF	3 (5)	4 (6)
Congestive heart failure	2 (3)	4 (6)
History of PCI	5 (8)	4 (6)
Current tobacco use	20 (32)	19 (31)
Clinical presentation		
STEMI	50 (81)	45 (73)
NSTEMI	7 (11)	8 (13)
UAP	5 (8)	9 (14)
Medications		
Statins	62 (100)	62 (100)
Atorvastatin	7 (11)	8 (13)
Rosuvastatin	53 (85)	51 (82)
Pitavastatin	1 (2)	1 (2)
Pravastatin	1 (2)	2 (3)
Fibrate	1 (2)	0 (0)
Ezetimibe	5 (8)	6 (10)
PCSK9 antibody therapy		
Evolocumab 140 mg every 2 weeks	28 (45)	NA
Evolocumab 420 mg every 4 weeks	12 (19)	NA
Alirocumab 75 mg every 2 weeks	21 (34)	NA
Alirocumab 150 mg every 4 weeks	1 (2)	NA
Anti-platelets		
Aspirin	60 (97)	60 (97)
Prasugrel	54 (87)	52 (84)
Clopidogrel	6 (10)	8 (13)
Anti-coagulants		
Warfarin	0 (0)	1 (2)
DOAC	2 (3)	3 (5)
ACE-I or ARB	45 (73)	46 (74)
β-blocker	38 (61)	28 (45)
Ca blocker	11 (18)	8 (13)
Laboratory data		
Hb, g/dL	13.9 ± 1.7	13.4 ± 1.7
Cr, mg/dL	0.9 ± 0.2	0.9 ± 0.3
LDL-C level, mg/dL	113.2 ± 32.8	111.3 ± 30.3
HDL-C level, mg/dL	45.0 ± 12.4	42.5 ± 12.0
Triglyceride level, mg/dL	137.2 ± 69.6	136.3 ± 77.5
Glucose, mg/dL	125.7 ± 29.4	127.0 ± 52.7
HbA1c, %	6.3 ± 0.6	6.3 ± 1.1
Echocardiographic data		
LVEDD, mm	46.4 ± 5.7	48.4 ± 6.1
LVESD, mm	31.1 ± 6.4	33.3 ± 6.2
EF, %	57.6 ± 10.0	56.2 ± 10.0

Data are shown as n (%) or means ± SD. Abbreviations: ACE, angiotensin-converting enzyme; AF, atrial fibrillation; ARB, angiotensin receptor blocker; BMI, body mass index; COPD, chronic obstructive pulmonary disease; Cr, creatinine; DOAC, direct oral anticoagulants; EF, ejection fraction. Hb, hemoglobin; HbA1c, hemoglobin A1c; HDL-C, high-density lipoprotein cholesterol; LDL-C, low-density lipoprotein cholesterol; LVEDD, left ventricular end-diastolic diameter; LVESD, left ventricular end-systolic diameter; NSTEMI, non-ST-segment elevation myocardial infarction; PAD, peripheral artery disease; PCI, percutaneous coronary intervention; PCSK9, proprotein convertase subtilisin-kexin type 9; STEMI, ST elevation myocardial infarction; UAP, unstable angina pectoris.

**Table 2:** Procedural characteristics.

	With PCSK9 Antibody Group (N = 62)	Without PCSK9 Antibody Group (N = 62)
Target lesion		
RCA	23 (37)	17 (27)
LMT	2 (3)	1 (2)
LAD	24 (39)	33 (53)
LCX	13 (21)	11 (18)
Stents		
CoCr-EES	27 (40)	25 (37)
Others	40 (60)	43 (63)
Multiple stents	5 (8)	8 (13)
Stent diameter, mm	3.1 ± 0.5	3.1 ± 0.5
Total number of stents	1.1 ± 0.3	1.1 ± 0.4
Total stent length, mm	26.0 ± 10.4	27.4 ± 13.2
Final TIMI III	53 (86)	58 (94)

Data are shown as n (%) or means ± SD. Abbreviations: EES, everolimus-eluting stent; LAD, left anterior descending artery; LCX, left circumflex artery; LMT, left main trunk; RCA, right coronary artery; TIMI, thrombolysis in myocardial infarction.

**Table 3:** Time points of laboratory test abnormalities and myalgia.

Event	With PCSK9 Antibody Group (N = 62)	Without PCSK9 Antibody Group (N = 62)	*p* Value
AST ≥ 3 upper limit of normal range			
1 month	0 (0)	0 (0)	1.0
3 months	0 (0)	0 (0)	1.0
12 months	1 (1.6)	0 (0)	1.0
ALT ≥ 3 upper limit of normal range			
1 month	0 (0)	0 (0)	1.0
3 months	0 (0)	0 (0)	1.0
12 months	2 (3.2)	0 (0)	0.496
CK ≥ 5 upper limit of normal range			
1 month	0 (0)	0 (0)	1.0
3 months	0 (0)	0 (0)	1.0
12 months	0 (0)	0 (0)	1.0
Myalgia			
12 months	1 (1.6)	0 (0)	1.0

Data are presented as n (%). Abbreviations: ALT, alanine aminotransferase; AST, aspartate aminotransferase; CK, creatine kinase.

**Table 4:** Serum LDL-C, HDL-C, and triglyceride levels at 1, 3, and 12 months.

	With PCSK9 Antibody Group (N = 62)	Without PCSK9 Antibody Group (N = 62)	*p* Value
1 month			
LDL-C, mg/dL	28.5 ± 14.6	80.5 ± 18.4	<0.001
HDL-C, mg/dL	53.2 ± 11.9	46.7 ± 11.8	0.003
Triglyceride, mg/dL	125.2 ± 60.9	145.1 ± 74.4	0.111
3 months			
LDL-C, mg/dL	32.8 ± 19.6	80.6 ± 23.3	<0.001
HDL-C, mg/dL	53.4 ± 12.2	48.9 ± 13.3	0.056
Triglyceride, mg/dL	128.9 ± 87.5	127.8 ± 66.7	0.942
12 months			
LDL-C, mg/dL	79.5 ± 19.6	80.2 ± 29.1	0.875
HDL-C, mg/dL	50.5 ± 12.9	49.4 ± 12.7	0.652
Triglyceride, mg/dL	148.0 ± 70.0	128.1 ± 63.8	0.109

Data are shown as means ± SD. Abbreviations: HDL-C, high-density lipoprotein cholesterol; LDL-C, low-density lipoprotein cholesterol.

## Data Availability

The data that support the findings of this study are available from the corresponding author upon reasonable request.

## Supplementary Materials

The following supporting information can be downloaded at: https://www.mdpi.com/article/10.3390/jcdd10050204/s1. Table S1. Potential adverse events which were not categorized in primary and secondary outcomes. The participating institutions and investigators of START-PCSK9 are listed in the Supplementary Materials.
