# Supplementary material for: Feasibility of Short-Term Aggressive Lipid-Lowering Therapy with the PCSK9 Antibody in Acute Coronary Syndrome
Source: J Cardiovasc Dev Dis. 2023 May 9;10(5):204. doi: 10.3390/jcdd10050204 (PMC10219227; doi:10.3390/jcdd10050204)
Supplement: Supplementary file 1 [file jcdd-10-00204-s001.zip › jcdd-2362276-supplementary.pdf]

Supplemental materials

Supplemental table S1. Potential adverse events which were not categorized in primary and secondary outcomes.

No.	Age (y)	Sex	Study group	Observation period	Details of adverse event	Causation with PCSK9 Ab treatment	Comments
1	73	Male	With PCSK9 Ab (Alirocumab)	1-month	Eosinophilic gastroenteritis	Unknown	
2	66	Male	With PCSK9 Ab (Evolocumab)	1-month	Skin rash	None	Caused by contrast medium injection
				3-month	Mild CK elevation	Unknown	CK level < 5 times of the upper limit of normal
3	77	Male	With PCSK9 Ab (Evolocumab)	1-month	Serum Cre elevation	None	Caused by oral ARB
4	84	Male	With PCSK9 Ab (Alirocumab)	12-month	Hospitalization due to bladder cancer	None	Known history of bladder cancer before initiating PCSK9 Ab
5	77	Male	With PCSK9 Ab (Evolocumab)	12-month	Worsening of CKD	None	Worsened at 1 year time point, PCSK9 Ab had already been stopped at 3 months
6	71	Female	Without PCSK9 Ab	1-month	Worsening of HF	None (non-PCSK9 Ab group)	Treated with oral diuretics, Hospitalization (-)
7	90	Female	Without PCSK9 Ab	1-month	Worsening of HF	None (non-PCSK9 Ab group)	Treated with oral diuretics, Hospitalization (-)
8	70	Male	Without PCSK9 Ab	1-month	Delayed cardiac tamponade	None (non-PCSK9 Ab group)	Due to oozing LV rupture caused by broad anterior MI
				1-month	Dry cough	None (non-PCSK9 Ab group)	Caused by oral ACE inhibitor
9	72	Male	Without PCSK9 Ab	3-month	Hemorrhagic gastritis	None (non-PCSK9 Ab group)	
10	48	Male	Without PCSK9 Ab	3-month	mild CK elevation	None (non-PCSK9 Ab group)	CK level < 5 times of the upper limit of normal
11	78	Male	Without PCSK9 Ab	12-month	Delirium	None (non-PCSK9 Ab group)	
12	82	Female	Without PCSK9 Ab	12-month	Ulcerative colitis	None (non-PCSK9 Ab group)	

13	73	Male	Without PCSK9 Ab	12-month	Pacemaker implantation due to sick sinus syndrome	None (non-PCSK9 Ab group)	
14	78	Male	Without PCSK9 Ab	12-month	Progression of interstitial pneumonia	None (non-PCSK9 Ab group)	
15	75	Female	Without PCSK9 Ab	12-month	Trauma (Femoral neck fracture)	None (non-PCSK9 Ab group)	

Abbreviations: Ab=antibody, ACE=angiotensin converting enzyme, ARB=angiotensin receptor blocker, CK=creatine kinase, CKD=chronic kidney disease, HF=heart failure, LV=left ventricle, MI=myocardial infarction, PCSK9=proprotein convertase subtilisin kexin-9

START-PCSK9 study investigators.

Investigators involved enrollment of at least 1 patient (124 patients enrolled overall).
Department of Cardiology, Iwata City Hospital: Kazusa Hayashi, Toshimasa Oda, Terumori Sato, Kimito Mizuno, Masataka Maruyama.
Department of Cardiology, Fujinomiya City Hospital: Kazuya Nakamura, Sadanori Takagi, Nobuyuki Wakahara.
Department of Cardiology, Kikugawa City Hospital: Yusuke Shirai, Hiromutsu Tominaga.
Department of Internal Medicine, Enshu Hospital: Daishi Nonaka, Kazuto Ohno.
Department of Cardiology, Shizuoka City Shizuoka Hospital: Hirofumi Sugiyama, Shigetaka Kageyama.
Department of Cardiology, Hamamatsu Red Cross Hospital: Hiroyuki Aoshima, Gaku Matsukura, Takahiro Kanda, Jun Tamura.
Department of Cardiology, Hamamatsu Medical Center: Kohei Sawazaki, Masakazu Kobayashi.
Department of Cardiology, Kosai General Hospital: Mamoru Nobuhara.
Internal Medicine III, Division of Cardiology, Hamamatsu University School of Medicine: Kyoko Unno, Yuichi Suzuki, Yutaro Kaneko, Takenori Ikoma, Keitaro Akita, Tomoaki Sakakibara, Ryota Sato, Keisuke Iguchi, Satoshi Mogi.
